# How to Improve Research Funding in Academia? Lessons From the COVID-19 Crisis

**DOI:** 10.3389/frma.2022.777781

**Published:** 2022-03-18

**Authors:** Vlasta Sikimić

**Affiliations:** Weizsäcker Center, University of Tübingen, Tübingen, Germany

**Keywords:** public funding, academic misconduct, elitism, life sciences, COVID-19

## Introduction

The current COVID-19 crisis has put both public and private funding of life sciences in the spotlight. One of the most frequent critiques of the scientific research conducted in industry is that researchers working for companies lack intellectual freedom. Moreover, from the perspective of the general public, industry research is always questioned because monetary interests might influence it. Sponsorship bias—a tendency of researchers working in the private sector to align their results with the interest of their funders—has been widely discussed in philosophy of science (e.g., Holman and Elliott, 2018; Leefmann, 2021). Some authors even go as far as opposing intellectual property in life sciences (Brown, 2008). Having all this in mind, epistemic trust in research conducted by companies is often lacking. However, it is questionable whether the academic sector alone, in its current state, can appropriately respond to global challenges. I argue that academic research requires substantial restructuring as similar objections can be raised both in the case of research done by academic institutions and in industry. Additionally, there are specific dangers connected with the current academic system such as elitism in science that are epistemically harmful. Though similar tendencies can also be detected in industry, academia has its own outdated rules that are reflected in its current culture.

It is important to note that not only academic institutions are publicly funded. Industry in certain contexts is also funded publicly, e.g., Moderna received almost one billion dollars from public sources for the development of its COVID-19 vaccine (Hussey, 2020). Different research schemes work better in certain contexts, but worse in others. In this sense, responsible science funding should be context-oriented.

When it comes to vaccine development, we witnessed many different funding approaches. For example, the Sputnik V vaccine was developed by a governmental institution. On the other end of the spectrum, the development of the BioNTech-Pfizer vaccine was mainly supported by pre-orders. Interestingly, the BioNTech-Pfizer collaboration even decided against taking funds from the US government to avoid the associated bureaucracy. Sinovac is another example for a private company that developed a COVID-19 vaccine without governmental funds. In the middle, we see public-private partnerships such as the joint-venture Sinopharm, the collaboration between the University of Oxford and AstraZeneca, and Moderna, a private company that received millions of dollars and logistic support from the US government.

In the context of mixed funding, it makes sense to ask whether certain academic institutions also support private interests. Moreover, publicly funded academic institutions might still have interests on their own, for instance building a reputation, being competitive, and financially profiting from that. From the perspective of individual researchers, the highly competitive nature of research in academia is fruitful ground for academic misconduct (e.g., Cartwright and Menezes, 2014). Furthermore, the publish or perish culture and limited contracts often motivate researchers to switch for a position in industry in which such existential pressures are not present (cf. Hayter and Parker, 2019).

There is a serious concern that academic prestige and elitism in both publicly and privately funded academic institutions have severe negative epistemic consequences. By elitist nature of science, I mean a broadly understood social construct where researchers with privileged backgrounds are favored over others. This extends to scientists belonging to a specific research institution, gender, origin, career stage, and other privileged groups. In this context, elitism affects both academics from the Global South and the ones employed by less prominent institutions in Western countries. As a result of elitism, the contribution of these researchers is not given equal weight as the input from the ones working in more famous, older or richer institutions.

During the pandemic, highly effective vaccines were developed in many different countries, including Russia and China. Moreover, we have to fight the pandemic in every country and every country needs the capacities to diagnose the disease, the experts to advise the government, and the ability to participate in clinical studies and vaccination campaigns. This emphasizes the need for epistemic decolonization as a prerequisite for a globalized academic effort.

Finally, the transparency of both academic and industry-related results is the key to building the necessary epistemic trust in science. This transparency is related both to the research data and the replicability of the results, as well as to the proper communication with the general public. A critical perspective is a necessary corrective requirement to make responsible scientific decisions and future improvements.

I will raise three arguments relevant for this debate. Firstly, research and development of COVID-19 vaccines is organized in various ways and most often involves a mixed funding approach involving public and private sponsors. Secondly, when assessing the epistemic consequences of mixed funding approaches a focus on industry sponsorship is one-sided. One also needs to take into account the non-epistemic interests of publicly funded research institutions and individual researchers. As I will point out, the working conditions for researchers in academia pose a constant threat to good scientific practice. Finally, I will argue that an attitude of elitism in both public and private research institutions and practices of epistemic colonization are major obstacles for reaching optimal decisions with regard to global health threats.

## The Interplay Between Private and Public Funding

Public and private funding do not always follow objective criteria. In this section, I will discuss how government spending is frequently not correlated with disease burden, neither on the global nor the national level, and how public funding can be awarded or withdrawn based on non-objective evaluations. Furthermore, I will highlight how governmental export restrictions influence the distribution of vaccines and protective equipment. Finally, I will use vaccine manufacturing as an example where private resources are needed to address a public health emergency.

Especially within the healthcare sector, private funding and patents have been extensively criticized (e.g., Bekelman et al., 2003; Brown, 2008). The reasons for this are manifold. One aspect concerns the focus on diseases typically encountered in richer countries, such as cardiovascular diseases and cancer (Trouiller et al., 2002). Companies typically invest more money into diseases that promise the highest revenues. However, also public funding is not only driven by disease burden—neither from the national nor the global perspective. Gillum et al. (2011) analyzed NIH funding for the year 2006 and found that the disease burden in the US only explains about one-third of the funding. While, for example, Diabetes mellitus received more funding than explained by the disease burden, research on depression received less than expected. Hence, neither public nor private entities necessarily focus on the most relevant issues, but instead on their own agendas.

From the perspective of the COVID-19 crisis, it is interesting to note that vaccines belong to a significantly underfunded category. In 2000, multinational vaccine companies invested <1 billion in the research and development of vaccines, which is <3% of their spending on pharmaceuticals (Régnier and Huels, 2013).

The objectivity of publicly funded science can be influenced by pressure to serve private interests, while academic institutions adopt cultures from the private sector (Azmanova, 2020). Even the selection of projects can already be skewed toward industry. To increase the (direct) applicability of research, some funding schemes require the involvement of private companies. Azmanova (2020) uses the example of Horizon 2020 to show that such programs do not only offload the investment risks to the society, while resulting patents are owned by companies, but also allow them to steer the research direction. In addition to influencing research agendas, some companies also manipulate the scientific discourse. For example, Monsanto sponsored ghostwriting in toxicology journals, influencing the opinion on its herbicide glyphosate (McHenry, 2018). As a long-term result, public trust in science gets challenged.

Different types of pressure can negatively influence the objectivity of researchers during the scientific process. For example, the evaluation of researchers based on publications, citations, and grants in combination with short-term contracts can negatively affect academic freedom (Zimmer, 2015). In addition, public funding holds sufficient examples of political interference into research agendas. Recently, the NIH canceled a program studying coronaviruses which was ongoing since 2014 due to political pressure (Rosenthal et al., 2020).

Currently, the standard division between research done in academia and industry is that applied research is done by industry while the foundational questions are tackled by academics. Public funding for research and development contributes up to two-thirds of the costs for developing drugs (Annett, 2021). In the future, to increase the robustness of the system and the possibility to develop medications cheaper and faster in the face of new challenges, more applied research could be done in academia. Finally, whether the development of infrastructure for mass drug production and their distribution should be publicly funded remains a question for political theory.

It is important to distinguish between research conducted in academia and research conducted in industry and to keep in mind that this distinction is not equivalent to the distinction between public and private funding. Companies can also be funded by governments (depending on the political system of the country), while many academic institutions are privately funded. Furthermore, scientists working in academia funded by the public sector sometimes also receive grants from private companies or foundations. Thus, to the argument that privately funded researchers lack academic freedom because they either explicitly or implicitly depend on the interest of the investors (Bekelman et al., 2003) also applies to researchers working in academia who are funded by private sources. However, this argument will not hold for the researchers working in publicly funded companies.

During the COVID-19 crisis, the private sector has been perceived as harmful for the distribution of vaccines to the Global South, because the profit leaned toward the countries that were paying the most. This in turn results in human casualties, even greater inequalities between countries, and increased mutation potential of the virus which in turn affects the whole world. On the other hand, the distribution of fully publicly funded vaccines is also not based on the idea of equity and patents prevent production in other countries. The EU, India, and the US, and thereby most vaccine-developing countries, have imposed export restrictions on vaccines or ingredients (Ibrahim, 2021). In addition, only a few countries allow foreigners to be vaccinated, even in places where there is an abundance of vaccines. In order to overcome such problems, a shift in the international arena would need to happen, facilitating the transition from a self-centered competitive model to a collaborative model that promotes solidarity between countries. Thus, in this context life sciences research should be understood as a global endeavor.

In vaccine development during the COVID-19 pandemic, we witnessed collaborations from the private and public sphere, industry, and academia. One of the reasons is that the infrastructure for the production and distribution of large amounts of vaccine doses was provided by industries that have such resources and capacities. On the other hand, research and development of new drugs, vaccine techniques, and treatments might not be overly profitable from the perspective of big pharma companies that sometimes prefer to outsource these activities and buy tested products from smaller players.

## Is the Current Academic Setting Worse Than the Industrial One?

To understand what motivates researchers to move from academia to industry, one has to compare working conditions for highly educated workers such as life scientists in both. A recent survey with more than 3,000 researchers as participants revealed a much higher satisfaction of scientists working in industry than in academia (Woolston, 2021). Researchers from industry feel more optimistic about their careers. A difference in job satisfaction was also detected between participants with permanent jobs and the ones with fixed-term contracts (Woolston, 2021). Industry offers well-paid permanent positions which allow for security, future planning, and general stability in life. In academia, temporary contracts are dominant and often one cannot even choose a place of living easily. Additionally, since there are more temporary junior than permanent senior positions for life scientists in academia, many scholars will not get the opportunity to become professors (Hayter and Parker, 2019). The general atmosphere in academia is highly competitive and this leads to numerous problems. The academic culture is often described as masculine (Gonsalves et al., 2016), and the reduced promotion of females reflects in the so-called leaky pipeline. The leaky pipeline means that women get less frequently promoted into higher positions and more frequently leave academia (Blickenstaff, 2005).

Some of the implicit rules of academia reflect its traditional, masculine, and retrograde setup in which junior researchers are dependent on their supervisors, success is not always objectively attributed, traditional elitist discrimination is in place, etc. In academia (self)exploitation of researchers is frequently justified with the love for science and the freedom it promises (Busso and Rivetti, 2014; Woolston, 2021). Furthermore, Zheng (2018) identified the idea that academics work for their own reward as a myth primarily sustained by the lucky few with stable employment. For these reasons, young researchers in life sciences often turn from academia to industry (cf. Hayter and Parker, 2019). In the private sector profit is the main parameter that drives success, while in academia early-career researchers are dependent on the evaluation of their group leaders. If the group leader is problematic, e.g., exploitative or oppressive, it might be difficult to make any change in the academic setting where senior researchers are often hard to suspend or replace. In contrast, private companies usually employ a professional HR and monitor the performance of the supervisors.

The pressure to publish, spearheaded by job uncertainty, can lead to violations of research standards. Bibliographic data is often used as the most important parameter for evaluating scientific and academic achievements. Thus, scholarships, jobs, academic positions, and research funding are dependent on the publication record (e.g., Bird, 2006; Bedeian et al., 2009). In order to meet the very high publishing standards, scientists might turn to different types of violations of research conduct, such as publishing insufficiently supported results, double publishing, self-plagiarism, producing “minimal publishable unit” (Neill, 2008), etc. Tijdink et al. (2014) showed that publication pressure among European medical scientists strongly correlates with scientific misconduct. Moreover, 72% of the participants in the study evaluated the publication pressure as too high, while 15% of them confessed that they had participated in the fabrication, falsification, or manipulation of data in the previous 3 years. It should, of course, be noted that academic fraud is not limited to junior researchers with insecure job perspectives. Based on focus-group discussions with more than 50 scientists, Anderson et al. (2007) identified competition and the “winner-take-all-approach” as a driver for scientific misconduct. Fang et al. (2013) analyzed the demographic data from the United States Office of Research Integrity which oversees misconduct investigations. Among the 228 scientists who committed misconduct, males were overrepresented, particular among faculty members.

Le Maux et al. (2019) used formal modeling to show that the monetary award of publishing in an influential journal increases academic misconduct. They concluded that if one wants to positively influence scientific output, publications in lower-ranked journals should also be rewarded.

One of the well-known examples of scientific misconduct with severe impact on society is a 1998 study by Wakefield and his co-authors published in *The Lancet*. The study fraudulently reported an MMR vaccine-induced syndrome characterized by chronic gastrointestinal symptoms and autism (Flaherty, 2011; Godlee et al., 2011). After the study became known to the general public, parents' distrust in the vaccination program increased, causing more parents to refuse to vaccinate their children. Vaccination rates in the UK fell from 91% in 1998 to <80% in 2003 (Flaherty, 2011). As a result of a lack of immunity, measles outbreaks began to occur in the UK. This example shows that academic research can also have severe negative consequences on trust in science.

The paper was based on 12 children, who were selected in favor of families reporting an association between autism and the MMR vaccine, and relied on parental recall and beliefs (Flaherty, 2011; Godlee et al., 2011). Furthermore, Wakefield received ~$670 000 from attorneys of families allegedly harmed by vaccines and held a patent on a new vaccine (Flaherty, 2011). Wakefield and his team were found to be in a conflict of interest, while the data presented in the publication were considered fraudulent (Godlee et al., 2011). Even though many studies refute the association between autism and MMR (e.g., Taylor et al., 1999; Farrington et al., 2001; Takahashi et al., 2003), and the article itself was withdrawn in 2010, the impact of Wakefield's article is still present because the public confidence in the safety of vaccination has been compromised. One reason for the large impact of Wakefield's study, which was immediately criticized by the scientific community, was his marketing strategy, involving a public relations company and press conferences (Irzik and Kurtulmus, 2019). Moreover, about half of the media coverage about the alleged link between the MMR vaccine and autism gave equal weight to his claims and the scientific consensus, while about one third only reported his claims (Irzik and Kurtulmus, 2019). In combination with his authority as a doctor at a respected hospital and the fame of one of the most influential medical journals, all these factors all contributed to the impact of his claims.

There are also indirect and long-term consequences of this publication reflected in the loss of confidence in the epistemic authority of scientists. Moreover, not only may the general public lose trust in the epistemic authority of scientists, but other scientists may also lose trust in their peers. Wakefield's and colleagues' publication can be considered the individual case of scientific misconduct with the largest negative impact on public health. This enormous impact is partially caused by the image of academic researchers as objective and impartial observers.

In order to decrease academic misconduct, one should work on the improvements of work conditions in academia and offer permanent contracts comparable to those in industry. The creative process in science cannot be easily stimulated externally, but certain conditions influence the research output. Directing funds into a system that promotes research quality and academic honesty instead of hyperproduction and competitiveness would make academia more apt to respond to global challenges. This also includes more opportunities for researchers from less-known research centers by financing their projects.

The importance of including researchers from all countries in the scientific discourse together with their diverse perspectives becomes particularly salient in the context of global challenges. The elitist nature of academia makes epistemic inclusion of underprivileged groups more difficult. While funding can promote international collaboration among researchers, elitism remains a challenge that needs to be overcome by changing the academic culture.

## Dangers of Elitism in Science

The danger of the elitist approach in science is that researchers from less famous scientific communities are discriminated. This can also have strong practical consequences. As part of the worldwide immunization during the COVID-19 pandemic, we witnessed that the European Medicines Agency (EMA) did not approve all the vaccines that the World Health Organization (WHO) approved, resulting in confusing policies, increased sense of inequality, skepticism toward certain vaccines, etc. In this way important results from less “prestigious” academic institutions may get hindered, fewer funds will be allocated to them, which again would enhance the current epistemic colonization. For example, in some European countries, foreigners immunized with all WHO approved vaccines are considered vaccinated, while others only accept EMA approved vaccines.

Epistemic colonization stands for imposing dominant epistemic attitudes and solutions to parts of the world which would originally have different epistemic tendencies. Mitova explains that the background assumption of epistemic colonization is that there can only be one best approach to science. The Global North, under this pretext, prescribes what counts as a rational and scientific solution disregarding the differences in the cultural context (Mitova, 2020). An important reason for fostering science in all institutions in a manner of equity is that for some solutions it is important to know local circumstances. For example, in 2018, a person was killed by his neighbors after he received a vaccine against Ebola because his neighbors thought he was infectious and would bring Ebola to their area. To avoid this risk, the WHO changed its vaccination strategy and gave people the option to be vaccinated in neighboring towns (Maxmen, 2019). The epistemic solutions and healthcare measures thus cannot just be imposed without prior knowledge of local circumstances. Collaborating with the local community and considering the local knowledge and beliefs in decision making is an important part of epistemic decolonization and can improve the effectiveness of public health care measures. For example, Liaw et al. (2011) established the utilization of local knowledge and community engagement as prerequisites for chronic disease care of Aborigine Australians. Similarly, the 2014 Lancet Commission on Culture and Health identified the systematic neglect of culture as the biggest barrier to the advancement of health care (Napier et al., 2014).

To respond to global challenges, we need a coordinated global strategy which requires an inclusive and stimulating environment with a non-elitist approach. As a response to epistemic injustice, Anderson (2012) argues in favor of equality as a virtue of institutions. This should be strengthened by the request for equity which means supporting marginalized groups with positive actions. In science, this means trust, respect, and financial support for researchers irrespective of their country of origin and taking affirmative actions when necessary.

## Conclusions

Though private funding of life sciences has been criticized as epistemically vulnerable and rightly so, I pointed out that simply turning to public funding of academia will not solve all the problems such as academic misconduct, biases in science, and its elitist nature. On the contrary, research in academia would benefit from significant restructuring to deal with global challenges that require fast solutions. Though privately funded bodies can have selfish incentives, countries themselves as fund providers can be governed by their egoistic motives. Moreover, in order to make academia more epistemically efficient, funding that would allow for permanent contracts for early-career academics would be beneficial. Since global challenges require coordinated action from all over the world and since science is a collaborative process, decreasing the elitist nature of science through funding diverse research from less known countries and institutions would bring positive results. These points were already known in epistemology of science, but the COVID-19 pandemic made them even more prominent and urgent.

Some of the ways of increasing the diversity in academia and strengthening international ties are funding schemes that promote global collaborations. These collaborations need to be constructed in a socially and epistemically just manner so that researchers from the Global South can take lead in projects instead of having marginal roles, e.g., data collection (Koskinen and Rolin, 2021). It is especially important to foster an inclusive academic environment where researchers from the Global South get fair acknowledgment and empowerment. Finally, funding agencies should promote a different academic culture which will allow researchers from currently underprivileged groups to be equally represented and flourish over time.

## Author Contributions

The author confirms being the sole contributor of this work and has approved it for publication.

## Conflict of Interest

The author declares that the research was conducted in the absence of any commercial or financial relationships that could be construed as a potential conflict of interest.

## Publisher's Note

All claims expressed in this article are solely those of the authors and do not necessarily represent those of their affiliated organizations, or those of the publisher, the editors and the reviewers. Any product that may be evaluated in this article, or claim that may be made by its manufacturer, is not guaranteed or endorsed by the publisher.
